# Keratoplasty to restore vision in trachomatous corneal opacity: A literature review

**DOI:** 10.1371/journal.pntd.0012535

**Published:** 2024-11-18

**Authors:** Iain Davidson, Matthew J. Burton, Noopur Gupta, Amir B. Kello, Jennifer Rose-Nussbaumer, Anthony W. Solomon

**Affiliations:** 1 Tennent Institute of Ophthalmology, NHS Greater Glasgow & Clyde, Glasgow, United Kingdom; 2 International Centre for Eye Health, London School of Hygiene & Tropical Medicine, London, United Kingdom; 3 Cornea, Cataract & Refractive Surgery Services, Dr. Rajendra Prasad Centre for Ophthalmic Sciences, All India Institutes of Medical Sciences, New Delhi, India; 4 World Health Organization Regional Office for Africa, Brazzaville, Republic of Congo; 5 Francis I. Proctor Foundation, University of California, San Francisco, San Francisco, California, United States of America; 6 Department of Ophthalmology, University of California, San Francisco, San Francisco, California, United States of America; 7 Global Neglected Tropical Diseases Programme, World Health Organization, Geneva, Switzerland; The Carter Center, UNITED STATES OF AMERICA

## Abstract

**Background:**

Trachoma is the leading infectious cause of blindness. Patients with trachomatous corneal opacity (TCO) are traditionally considered high-risk cases for graft failure. However, anecdotal evidence suggests that corneal transplantation may restore vision in such individuals. We wanted to review the available evidence for keratoplasty outcomes in TCO.

**Methods:**

A literature search of PubMed, MEDLINE, and Web of Science was performed using the search terms “trachoma* AND (keratoplasty OR cornea* transplant*)”. The search was restricted to studies published between 1 January 1992 and 12 October 2022. All types of prospective and retrospective study designs reporting outcomes of keratoplasty in trachoma were included. The primary outcome assessed was rate of graft survival in patients with TCO who received keratoplasty. Secondary outcomes were postoperative best corrected visual acuity (BCVA) and graft rejection rates.

**Results:**

Seven studies met our inclusion criteria. None were prospective trials; 215/302 grafts (71%) were clear at final follow-up. There was significant variability between studies in the reporting of patient characteristics, follow-up, complications, and outcomes. In data on penetrating keratoplasty (PKP), graft survival at final follow-up was observed in 161/195 eyes (83%). Studies assessing lamellar keratoplasty (LKP) reported graft survival in 18/20 eyes (90%). Rejection episodes were reported in 31/167 (19%) eyes managed with PKP and 0 of 20 eyes managed with LKP. Of 163 eyes, preoperative BCVA was ≤counting fingers in 76% and ≤6/60 in 91%. A postoperative BCVA of >6/60 was achieved in 63% of eyes.

**Conclusions:**

There is a paucity of evidence supporting keratoplasty in TCO. However, it may hold visual rehabilitation promise for people whose needs have to date been largely ignored. More structured reporting of outcomes from centres which perform keratoplasty in TCO and a well-designed prospective study would be valuable additions to the literature.

## Introduction

Trachoma is the leading infectious cause of blindness [1]. The infectious agent, serovars A-C of *Chlamydia trachomatis*, is transmitted within ocular and nasal secretions [2]. Active trachoma is characterised by an inflammatory keratoconjunctivitis [3]. Recurrent episodes of infection resulting in severe inflammation can progress to cicatricial changes and scarring of the upper tarsal conjunctivae, resulting in entropion and trichiasis [4]. Tear film instability (caused by goblet cell destruction from conjunctival cicatrisation) and the presence of lid abnormalities can contribute to a dry ocular surface. The combination of trichiasis and dry eye may lead to recurrent corneal erosions which, along with secondary bacterial infection, produce trachomatous corneal opacity (TCO) and accompanying visual impairment or blindness [5,6].

Trachoma has largely disappeared from industrialised countries, but is responsible for the visual impairment of approximately 1.9 million people in the world’s poorest communities [3,7]. The World Health Organization (WHO) recommends a package of interventions known as the “SAFE” strategy (surgery, antibiotics, facial cleanliness, and environmental improvement) to correct trichiasis and reduce *C*. *trachomatis* transmission, in order to eliminate trachoma as a public health problem [8].

Although the SAFE strategy provides a comprehensive approach towards prevention of visual impairment from trachoma, it does not address the management of those with existing visual impairment from advanced disease. While trachoma-related visual disability in a population will eventually disappear following the elimination of trachoma as a public health problem [9], the visual impairment of those living with TCO is a significant burden to individual patients and their support networks [10].

In many health systems, patients with TCO are not considered candidates for keratoplasty due to assumptions that ocular pathology (including tear film changes, lid deformation, and corneal vascularisation) would impair graft survival [11]. However, anecdotal evidence suggests that keratoplasty may restore vision in such individuals. Recent progress in appropriate case selection, surgical techniques, and eye care infrastructure in endemic areas may contribute to improved graft survival.

This review aims to assess the available evidence of outcomes of corneal transplantation in TCO. If there is evidence to support it as a viable therapeutic option, corneal transplantation may have a significant role in reducing the future burden of trachoma-related visual impairment and may help policy makers re-evaluate a potentially treatable cause of blindness.

## Methods

References were identified through searches of PubMed, MEDLINE, and Web of Science using the search terms “trachoma* AND (keratoplasty OR cornea* transplant*)”. Because we expected there to be few published randomised control trials, all types of study designs reporting outcomes of keratoplasty in trachoma over any follow-up duration were included. No language restrictions were imposed. We limited our searches to studies published between 1 January 1992 and 12 October 2022 (our search date). It was the opinion of the research team that advances in surgical techniques and technology beyond this timeframe would limit the value of older studies. Reference lists of selected papers were also searched to identify additional studies.

The primary outcome assessed was rate of graft survival (defined as a clear graft at final follow-up) in patients with TCO who received keratoplasty. High heterogeneity was expected in the reporting of secondary outcomes, but we also assessed postoperative changes to best corrected visual acuity (BCVA) and rejection rates associated with keratoplasty where possible.

Two authors independently reviewed the abstracts of papers included in the search results to select papers for full-text review. Any papers selected by either author were included. The full text of each selected paper was scrutinised to determine whether the subjects meet inclusion criteria. Data extracted were: number of patients; patients’ ages and gender; rate of graft survival; operative technique; differences between preoperative and postoperative BCVA; incidence of graft rejection.

We calculated proportions of graft survival, rates of rejection, and differences in preoperative and postoperative BCVA. Subgroup analysis of reported outcomes in penetrating keratoplasty (PKP) and lamellar keratoplasty (LKP) was performed. Due to the heterogeneity of the data no formal meta-analysis was undertaken.

## Results

### Selection and identification of studies

Our searches identified 54 publications. One article was excluded due to duplication, meaning 53 separate articles were screened by our authors. Screening of article titles and abstracts with a low threshold for inclusion identified 14 articles that may have reported keratoplasty in trachoma and were subject for full-text review. Of these, 8 reported outcomes of corneal transplant in trachoma. One study was excluded as it did not differentiate between trachomatous and other postinfectious corneal opacity in its analyses [12].

### Characteristics of included studies

All included studies were retrospective and were conducted between 1994 and 2018 in Israel, Kenya, Türkiye, Japan, Saudi Arabia, the Islamic Republic of Iran, and India (Table 1). Three were large studies reviewing keratoplasty for all indications in their respective catchment areas, with subgroup analyses for trachoma, and sample sizes of 7, 45, and 87 eyes [13–15].

**Table 1:** Included studies and graft survival at final follow-up.

Study	Year	Setting	Number of eyes	Procedure	Graft survival	Mean follow-up in months (range)	Trachoma-specific study	Individual outcomes available
De Cock	1994	Israel	45	PKP	39 (87%)	NR	No	No
Yorston	1996	Kenya	7	PKP	6 (86%)	NR	No	No
Kocak-Midillioglu	1999	Türkiye	16	PKP	14 (88%)	26 (14–61)	Yes	Yes
Shimmura	2007	Japan	3	LKP	2 (67%)	16 (13–18)	No	Yes
Al-Fawaz	2008	Saudi Arabia	127	PKP	102 (80%)	42 (3–114)	Yes	Yes
Zare	2012	Islamic Republic of Iran	87	PKP+LKP	36 (41%)	NR	No	No
Sharma	2012	India	17	LKP	16 (94%)	16 (12–54)	Yes	Yes
Total			302		215 (71%)		3 (43%)	4 (57%)

PKP, penetrating keratoplasty; LKP, lamellar keratoplasty; NR, not reported.

Three studies specifically assessed outcomes of keratoplasty in trachoma, with sample sizes of 16, 17, and 127 eyes [5,9,16]. One study described the outcomes of 11 eyes which underwent keratoplasty for severe ocular surface disease, of which 3 had TCO [17].

Two patients in the study by Kocak-Midillioglu and colleagues [16] and 1 patient in the study by Shimmura and colleagues [17] received bilateral transplantation; otherwise, all cases were unilateral where reported. Individual patient characteristics, visual outcomes, and duration of follow-up were reported in these 4 articles only.

Four studies exclusively reported outcomes of PKP, and 2 reported outcomes of LKP. Zare and colleagues [15] reported on both types of transplants but did not differentiate between their outcomes in a subgroup analysis, so we are unable to include the data from that paper in our own subgroup analysis.

### Graft survival

In total, 215 (71%) of 302 grafts were clear at final follow-up. High heterogeneity was observed in the reporting of follow-up duration and frequency. Such data were only reported by Kocak-Midillioglu (26 months, range 14 to 61) [16], Shimmura and colleagues (16 months, range 13 to 18) [17], Al-Fawaz (42 months, range 3 to 114) [9], and Sharma and colleagues (16 months, range 12 to 34) [5]. Where reported, frequency of follow-up gradually tapered throughout the postoperative period but varied according to local protocol. For example, Sharma and colleagues [5] reported daily follow-up until discharge and then at 1 week, 1, 3, and 6 months and yearly subsequently. Mean or median number of follow-up visits was not reported in any study. There was no trachoma-specific follow-up frequency or duration reported in the studies which assessed keratoplasty for all indications. The timing of postoperative complications was not reported in any study.

The study by Shimmura and colleagues [17] reported that conjunctivalisation of 2 grafts was observed at final follow-up. It was unclear whether the authors considered these grafts to have failed, and despite contacting the authors for their elaboration, we did not receive a reply. As conjunctivalisation is not graft failure per se, we inferred that one of the grafts was successful due to an excellent improvement in visual acuity from counting fingers (CF) preoperatively to 6/15 at final follow-up (Table 2).

**Table 2:** Reported patient characteristics and distance visual acuity.

Study	Number of eyes	Number of female patients	Mean age (range)	Pre-op BCVA ≤ 6/60	Pre-op BCVA ≤ CF	Post-op BCVA ≥6/48	Number of rejection episodes
De Cock	45	NR	NR	NR	NR	NR	NR
Yorston	7	NR	NR	NR	NR	NR	4 (57%)
Kocak-Midillioglu	16	5 (31%)	64 (51–78)	16 (100%)	16 (100%)	13 (81%)	5 (31%)
Shimmura	3	NR	NR	3 (100%)	3 (100%)	1 (33%)	0 (0%)
Al-Fawaz	127	66 (52%)	65 (40–90)	115 (91%)	105 (83%)	72 (57%)	22 (17%)
Zare	87	NR	NR	NR	NR	NR	NR
Sharma	17	13 (76%)	50 (17–75)	15 (88%)	0 (0%)	17 (100%)	0 (0%)
Total	302			149/163 (91%)	124/163 (76%)	103/163 (63%)	31 (18%)

NR, not reported; BCVA, best-corrected visual acuity; CF, counting fingers.

Among eyes managed with PKP, graft survival at final follow-up was observed in 161 (83%) of 195 eyes. The 2 studies assessing LKP reported graft survival in 18 (90%) of 20 eyes. Zare and colleagues [15] was excluded from this analysis as it did not differentiate outcomes following PKP and LKP, as noted above. The authors were contacted for elaboration but we did not receive a reply.

### Visual acuity

Pre- and postoperative BCVA was reported for 163 eyes across 4 studies (Table 2). The studies of Kocak-Midillioglu and colleagues [16], Shimmura and colleagues [17], and Sharma and colleagues [5] included data for each patient. However, the larger study by Al-Fawaz and colleagues [9] reported visual acuity in the following groups: 20/40 or better, 20/50–20/160, 20/200–20/800, CF, hand movements (HM), light perception (LP), and no light perception (NLP). Comparisons of pre- and postoperative BCVA for individual patients was therefore not possible. In the present study, it was determined that ≥6/48 was an appropriate cut off for visual acuity to ensure all available data could be included in our results.

Overall, an improvement in visual acuity following transplantation was observed. Preoperative BCVA was ≤6/60 in 149 eyes (91%), and a postoperative BCVA of ≥6/48 was achieved in 103 eyes (63%); 25 eyes (15%) achieved a postoperative BCVA of 6/12 or better.

### Graft rejection

All studies except for De Cock [13] and Zare and colleagues [15] described rates of graft rejection. In the studies of Yorston and colleagues [14], Kocak-Midillioglu and colleagues [16] and Al-Fawaz and colleagues [9], there were 31 (21%) reported rejection episodes in 150 eyes managed with PKP. The specific timeframe of rejection episodes was not reported in any study. None of the 20 eyes undergoing LKP had a documented rejection episode.

## Discussion

### Evidence and rationale for keratoplasty in TCO

The current review suggests that keratoplasty may be effective in TCO; however, it has also highlighted the paucity of available evidence. Only 8 papers met our broad inclusion criteria, and all were retrospective analyses. Limited information was available in these publications regarding preoperative issues such as severity of conjunctival scarring, severity of ocular surface and adnexal disease, or presence/extent of neovascularisation, and we found high heterogeneity of follow-up duration and reporting of visual outcomes. One large retrospective analysis from Ethiopia had to be excluded as it did not differentiate between trachoma and other causes of postinfectious corneal opacity, which was essential for our purposes [12]. While the study from Japan by Shimmura and colleagues [17] reported complication rates and final phenotype, it was unclear whether the 2 grafts with conjunctivalisation were considered by the authors to have been successful.

Trachomatous keratopathy remains a significant cause of visual impairment in lower income countries, disproportionately affecting the poorest and least well-served members of the world’s population. The presence of corneal vascularisation, reduced tear film production and instability, ocular surface disease, and lid abnormalities mean patients with TCO are considered high-risk cases for transplantation failure [5], and trachoma is still generally considered a cause of “irreversible” blindness [7]. However, the results of the present study suggest keratoplasty may be an appropriate therapeutic option for some patients.

The effective implementation of WHO’s SAFE strategy is reducing the burden of trachoma as a public health problem in most countries, and the prevalence of trachomatous visual impairment is declining due to a falling incidence of patients with TCO [9]. However, there remains a rationale for exploring keratoplasty in these individuals. Progressive development of eye care infrastructure, as illustrated by rising cataract surgery rates in Asia and Africa, have provided a base from which corneal transplant services have emerged [18]. In 1996, Yorston and colleagues [15] described the use of keratoplasty in an African setting, and an eye-bank has been operating in Ethiopia since 2003, increasing access to sight-restoring transplantation for residents of a country that bears half the world’s burden of active trachoma [12]. Ayalew and colleagues [12] reported that in Ethiopia between 2000 and 2013, trachoma/postinfectious corneal opacity was the leading indication for corneal transplant (141 of 321, 44%), followed by keratoconus (14%), corneal dystrophy (14%), pseudophakic/aphakic bullous keratopathy (9%), trauma (8%), active ulcer/burn/perforation (3%), and other indications (2%). They found superior graft survival at 2 years for keratoconus (100%) compared to trachoma/postinfectious corneal opacity (78%), corneal dystrophy (78%), pseudophakic/aphakic bullous keratopathy (83%), prior graft failure (56%), trauma (94%), active ulcer/burn/perforation (86%), and all other indications (75%). This study could not be included in the present analysis as they did not differentiate outcomes for trachoma and other causes of postinfectious corneal opacity.

### Factors affecting graft survival

There are multiple factors that have classically been attributed to poor graft survival in trachoma. Conjunctival scarring causes destruction of goblet cells and accessory lacrimal gland tissue, resulting in reduced tear film volume and stability, and can contribute to ocular surface disease [19]. This is also affected by lid contour abnormalities, particularly entropion, and associated trichiasis may cause mechanical damage to a host or donor cornea [20]. A fragile ocular surface is associated with bacterial keratitis, an important cause of graft failure. The presence of corneal neovascularisation is also recognised to have a deleterious effect on graft survival [21]. However, it is notable that the neovascularisation seen in trachoma is typically superficial, which is less strongly associated with lowered graft survival than deep vessels.

Authors of several included studies implied that appropriate case selection and management of extra-ocular factors can improve rates of graft survival. In their case series, Kocak-Midillioglu and colleagues [16] described the preoperative management of dry eyes, Meibomian gland disease, trichiasis, and entropion. Al-Fawaz and colleagues [9] alluded to the importance of careful case selection. They did not specify objective measures that determined patient suitability, but did comment on low rates of early and late epithelial defects, supporting their hypothesis of achieving well-controlled ocular surface disease prior to transplantation. Sharma and colleagues [5] performed punctal cautery on patients with a preoperative Schirmer value less than 10 mm and performed surgical correction of entropion at least 2 months prior to keratoplasty, following the recommendation of Monga and colleagues [20]. However, compared with a rate of 4% of late persistent epithelial defects (lasting over 14 days past the initial postoperative period) in Al-Fawaz and colleagues’ study [9], 35% of the eyes in Sharma and colleagues’ study [5] still developed persistent epithelial defects. These were treated with bandage contact lenses and amniotic membrane grafts, of which only one resulted in graft failure due to infection.

### Penetrating versus lamellar keratoplasty

In their study reporting the outcomes of LKP in trachoma, Sharma and colleagues [5] suggested that automated therapeutic LKP can successfully manage TCO. Graft survival at final follow-up was achieved in 94% of eyes, with one eye developing a graft infection secondary to persistent epithelial defect; 88% of eyes had a preoperative BCVA of ≤6/60, and 94% achieved a postoperative BCVA of 6/24 or better. Zare and colleagues [15] noted that during their study period that LKP became the treatment modality of choice for TCO, but did not report on the differences in graft survival between PKP and LKP subgroups in their series of 87 eyes. Graft survival rate in trachomatous eyes was relatively low (41%) in their study when compared to other indications such as keratoconus (89%), aphakic/pseudophakic bullous keratopathy (75%), non-herpetic corneal scar (69%), failed graft (47%), and active infectious ulcer (33%). Yorston and colleagues [14] also observed excellent graft survival in eyes with keratoconus (88%) compared to non-keratoconus grafts (65% overall, 86% in trachoma).

PKP was the dominant keratoplasty technique for all indications throughout the 20th century [22]. PKP was often preferred over a lamellar approach as it avoids the process of manual dissection, which was technically demanding and time-consuming, and an irregular graft-host interface may result in higher rates of postoperative astigmatism, scarring, and a poor visual outcome [23,24]. The development of new microkeratomes for refractive surgical procedures, such as laser in situ keratomileusis, has contributed to a preference for lamellar transplants where appropriate [25]. Automated keratomes have been demonstrated to be relatively quick, easy to use, and provide a high-quality cut, resulting in excellent visual outcomes in cases of mid- and anterior stromal scarring [24]. LKP is now generally accepted to have fewer intra- and postoperative complications than PKP given it is a “closed-sky” operation [26]. It also allows for the use of one donor lenticule for multiple transplantation of different corneal layers. In their series, Sharma and colleagues [5] reported that the anterior lenticule remaining after Descemet stripping automated endothelial keratoplasty was used as donor tissue for 4 eyes (24%). Superficial anterior lamellar keratoplasty (SALK), first described in 2003 and currently used for TCO in India, uses fibrin glue rather than sutures to secure the graft [27]. Sutures are associated with increased rates of microbial keratitis, graft failure, and postoperative astigmatism [28], so this sutureless approach may have a positive impact on graft survival, visual outcome, and the burden of follow-up for patients.

### Other challenges

Patients who undergo keratoplasty require regular follow-up, particularly in the early postoperative period. Rejection episodes and other postoperative complications occur with sufficient frequency that access to services and medication and willingness of the patient to engage with follow-up and treatment protocols are essential. Given TCO typically affects poorer communities, financial support, or subsidisation would likely be required in most instances. Final BCVA is affected by the timing of suture removal, which is itself a risk for graft infection and rejection [28,29], and the availability of refraction and glasses [9]. Requiring proximity to tertiary eye care for a short period after the operation may be a small price to pay, however: improvement to vision and the opportunity to regain independence are potentially life-altering for the patient and their circle of support [11].

Historically, there have been several barriers that have limited the role of corneal transplantation in reducing global blindness [18]. The majority of corneal blindness occurs in lower income settings with limited tissue availability, a lack of trained surgeons, inadequate accessibility to peri-operative care, and logistical and financial restrictions all constraining the ability of local health systems to develop cost-effective and logistically feasible corneal transplant services [18,30]. However, as global eye care infrastructure continues to improve and numbers of eye banks increase, transplantation is likely to play an important role in addressing corneal blindness.

### Reported outcomes versus global practice

Interestingly, several other papers identified in the literature search but not included in the present analysis list trachoma as a routine indication for keratoplasty, despite the absence of formal recommendations or prospective trial data [12,31–33]. Publications from Bahrain, Ethiopia, and India suggest trachoma as a leading indication for keratoplasty in their respective services [12,31–33]. This suggests there may be an imbalance between available data and current global practice. Given the paucity of available evidence, any reported outcomes regarding keratoplasty in TCO would be a valuable contribution to the literature. In particular, detailed reporting of coexisting preoperative pathology that may affect graft survival would be of use in determining which patients with TCO may be suitable candidates for corneal transplantation.

## Conclusion

As patients who become blind from trachoma are often found in poor and remote communities, the use of keratoplasty to address TCO could face practical barriers. However, there is a convincing argument that it has promise for visual rehabilitation for people whose needs have to date been largely ignored. Any reporting of outcomes from centres which perform keratoplasty in TCO would be a valuable addition to the literature. Even more importantly, a well-designed prospective study that analyses the important factors around case selection and formally estimates the incidence of good outcomes over the longer term could help policy makers re-evaluate a potentially treatable cause of blindness.
